# Use of integrin alpha 6 transcripts in a stool mRNA assay for the detection of colorectal cancers at curable stages

**DOI:** 10.18632/oncotarget.7407

**Published:** 2016-02-15

**Authors:** Jean-François Beaulieu, Elizabeth Herring, Shigeru Kanaoka, Éric Tremblay

**Affiliations:** ^1^ Laboratory of Intestinal Physiopathology, Faculty of Medicine and Health Sciences, Université de Sherbrooke, Sherbrooke, QC, Canada; ^2^ Department of Gastroenterology, Hamamatsu Medical Center, Hamamatsu, Japan

**Keywords:** colorectal cancer, adenomas, non-invasive screening, biomarker, mRNA

## Abstract

**Objective:**

An important criterion for colorectal cancer (CRC) screening is the ability to detect lesions at a curable stage. In the present study, we have assessed the integrin α6 subunit transcript (*ITGA6*) as part of a stool assay for the detection of colorectal lesions.

**Results:**

In comparison with control samples, *ITGA6* levels were found to be significantly increased at all stages (*P* < 0.01). Receiver operating characteristic analysis revealed areas under the curve of 0.89 for the prediction of CRC with 81% sensitivity and 88% specificity and of 0.90 for the prediction of advanced adenomas (Ad) with 75% sensitivity and 88% specificity. The *ITGA6A* variant was also found to be increased relative to *ITGA6* in stage II and III CRCs. Combining *ITGA6* with other selected transcripts and/or immunochemical fecal occult blood test (iFOBT) results further increased sensitivity and specificity for the detection of colorectal lesions.

**Patients and Methods:**

*ITGA6* detection used alone and under various combinations including detection of other mRNA markers and iFOBT was assessed on stool samples obtained from 175 patients (91 CRCs, 24 Ad and 60 healthy controls).

**Conclusions:**

These data confirm the usefulness and reliability of an mRNA stool assay for the detection of colorectal lesions. The validation of additional candidate genes and their analysis in multiplex qPCR represents a powerful and robust approach that can be combined with iFOBT results to improve the detection of colorectal lesions.

## INTRODUCTION

Colorectal cancer (CRC) remains an important cause of death in the Western world [1]. Because this cancer can be successfully treated before the occurrence of metastasis, early and efficient diagnosis is crucial [2–4]. The sensitivity of the immunochemical fecal occult blood test (iFOBT) has significantly improved over the last decade as a non-invasive method to screen for CRC [5–11]. Unfortunately, although advanced adenoma (Ad) detection could contribute to preventing the occurrence of CRC, iFOBT sensitivity for these precancerous lesions remains low [5–7, 12].

Other non-invasive methods based on the detection of CRC specific markers released by tumor cells exfoliated into the stools by genomic (stool DNA) or transcriptomic (stool RNA) approaches appear promising [3, 13–16]. The particular interest in the latter is that candidate genes can be selected on the basis of their predominant expression in tumor cells. The usefulness of detecting stool messenger RNAs (mRNAs) for CRC screening has been previously demonstrated [17, 18] even though mRNAs are considered to be less stable in stools than other components more favored recently, such as DNA and proteins or even microRNAs [3, 15, 16, 19]. Target instability may not necessarily be a significant weakness in a CRC stool screening test. Indeed, as shown by one of us testing various mRNAs, the factors that contribute to mRNA increase in stools of patients with CRC include tumor size and the number of exfoliated cells but not tumor location [20]. In fact, the high rates of exfoliation of epithelial cells that remain at least partially preserved as suggested by the ability to detect long fragments of DNA and mRNA as well as even the cells themselves [20–25] support the approach.

Over-expression of integrin α6β4 in primary CRCs has been well documented by our group [26–28]. Most notably, the α6 subunit and particularly its α6A variant, which specifically promotes tumor cell proliferation, has been found to be up-regulated in more than 80% of CRCs at the transcript level [26, 27] suggesting that this integrin subunit could be a good candidate in a stool mRNA-based assay for the detection of colorectal lesions.

In this study, we tested this hypothesis by evaluating the usefulness of integrin α6 subunit transcript (*ITGA6*) detection alone and in combination with other transcripts from stool samples obtained from patients diagnosed with Ad and stage I to IV CRC. Furthermore, considering that tumor cell exfoliation and blood release in the stools may represent distinct phenomena, we also verified whether combining mRNA data with iFOBT results improves the detection of colorectal lesions.

## RESULTS

### *ITGA6* mRNA expression in stools

To evaluate *ITGA6* mRNA levels in stool samples of patients with diagnosed Ad or stage I to IV CRC, mRNA median levels were determined for each group as copy number and compared with the control sample group. Experiments first consisted of evaluating the uniformity of the preamplification reactions. For this purpose, qPCR for *ITGA6* and *B2M,* a reference gene [29], was performed on a subset of 47 samples including 10 Ad, 26 CRC and 11 controls on the same preamplified and non-preamplified cDNAs. Evaluation of the relative linearity between the two revealed a significant correlation for both *B2M* (r = 0.9457, *P* < 0.0001) and *ITGA6* (r = 0.7183, *P* < 0.0001) using nonparametric Spearman analysis confirming that both genes were amplified in a linear manner with the preamplification kit. Based on these data, we chose to use preamplified cDNA samples for the rest of the study.

**Figure 1:**
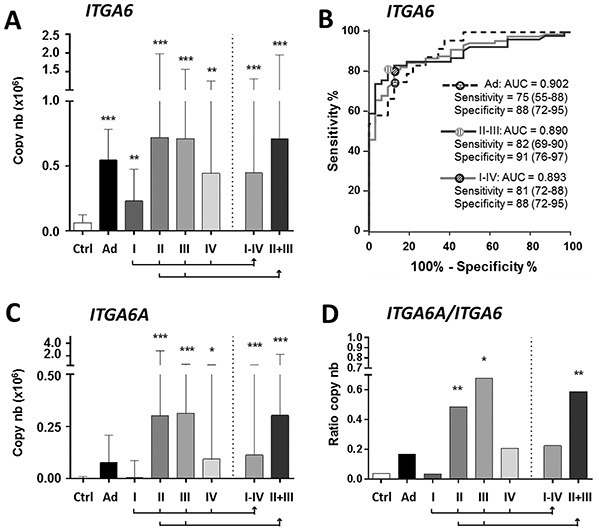
Detection of *ITGA6* in stool samples of controls and patients diagnosed with Ad and stage I to IV CRC **A.** For *ITGA6*, in comparison with controls (Ctrl), significant increases were observed in Ad and all CRC stages alone and in combination (I-IV and II+III). **B.** ROC curve analysis of ITGA6 detection in Ad, CRC II-III and CRC I-IV. Sensitivity and specificity are provided in % (95% CI). **C–D.** For the detection of *ITGA6A*, significant increases were observed for stage II-IV CRC individually and in combinations (I-IV and II-III) (C). *ITGA6A*/*ITGA6* ratios were found to be significantly increased for stage II and III CRC (D). Results in A, C and D are expressed as median (interquartile range) of copy number relative to control patients. * *P* < 0.05 to *** *P* < 0.001 using the Kruskal-Wallis test.

The median levels of *ITGA6* mRNA expression in stools of patients were found to be statistically significantly higher than in controls for all conditions including Ad (*P* < 0.001) and all CRC stages (*P* < 0.01) (Figure 1A). Receiver operating characteristic (ROC) analysis for *ITGA6* revealed an area under the curve (AUC) of 0.89 (*P* < 0.0001) for the prediction of stage I-IV CRC vs controls (Figure 1B) with 81% sensitivity and 88% specificity. The sensitivity and specificity of the fecal ITGA6 assay were 82% and 91% for stage II-III CRC and 75% and 88% for Ad.

Two isoforms of *ITGA6* are expressed in CRC primary tumors: *ITGA6B*, which encodes a variant that is associated with the quiescent state in the normal colon and *ITGA6A*, which encodes a pro-proliferative integrin α6A subunit that is up-regulated in a majority of CRC [26, 27]. Since *ITGA6A* up-regulation is responsible for the overall increase of *ITGA6* in CRC [27], overall *ITGA6* was detected in the mRNAs assays. However, analysis of *ITGA6A* using specific probes revealed statistically significant expression in stools of patients with stage II and III CRC (Figure 1C) while ITGA6A/ITGA6 ratios suggest that ITGA6A consists of approximately half of the total ITGA6 copy number in the stools of patients with stage II and III CRC (Figure 1D).

### *ITGA6* mRNAs vs iFOBT assay: the IF score

Another key result with the mRNA assay was the apparent independence relative to the iFOBT assay in the identification of patients with Ad or CRC. Indeed, as shown in Table 1, 20 of the 26 iFOBT negative CRCs and 13 of the 17 iFOBT negative Ads were found to be positive for *ITGA6.* To further investigate the combinatory use of the two approaches, we generated an index score combining the iFOBT and *ITGA6* results, the IF score. IF scores were calculated as the summation of the individual scores generated for *ITGA6* based on a method described previously by Ng et al [30] using the fold increase of each marker ranked on a scale of 0 to 3 on the basis of 3 cutoff values (lower cutoff corresponding to a sensitivity of 80%, medium cutoff corresponding to a specificity of 90% and higher cutoff corresponding to a specificity of 99%) and the iFOBT score (0 for negative and 3 for positive).

**Table 1:** Usefulness of *ITGA6* mRNA for identifying patients with adenomas or CRC displaying iFOBT negative test

Stage	n	iFOBT negative	ITGA6*	Rescue^2^
Ad	24	17	13	76%
CRC I	24	13	9	69%
CRC II	32	6	5	83%
CRC III	21	3	2	67%
CRC IV	13	4	4	100%
CRC total	91	26	20	77%

* Based on cutoff values selected to achieve 80% sensitivity

2 % of iFOBT negative samples identified as positive with *ITGA6*

**Figure 2:**
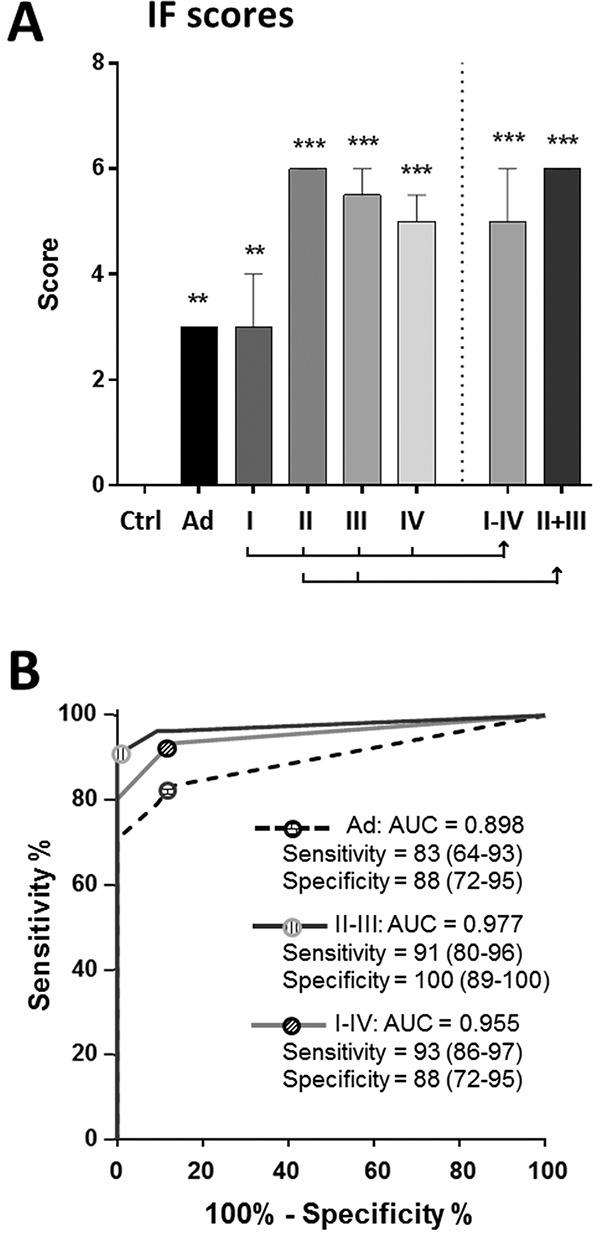
Combining ITGA6 detection with iFOBT: the IF score The IF score was calculated using an algorithm that combines ITGA6 and iFOBT. **A.** The results show a median score ≥ 3 for all types of lesions including Ad and stage I CRCs relative to controls; * *P* < 0.05 to *** *P* < 0.001 using the Kruskal-Wallis test. **B.** ROC curve analysis of the IF score for Ad, stage I-IV CRCs and stages II-III CRCs showing an overall increase in sensitivity and specificity for lesion detection as compared with ITGA6 alone (Figure 1B). Sensitivity and specificity are provided in % (95% CI).

IF score improved overall lesion detection in patients with Ad and CRC at all stages (Figure 2A). ROC analysis displayed an AUC of 0.96 for the prediction of stage I-IV CRC detection displaying 93% sensitivity and 88% specificity (Figure 2B). The combined use of ITGA6 and iFOBT also improved the detection of Ad and stage II-III CRC with sensitivities of 83% and 91%, respectively (Figure 2B).

### More target mRNAs: The IGM and IGMF scores

We then screened for additional mRNA markers. As proof of concept, a group of tested genes was selected on the basis of their reported expression in primary tumors in association with CRC recurrence risk (Oncotype DX©).

**Figure 3:**
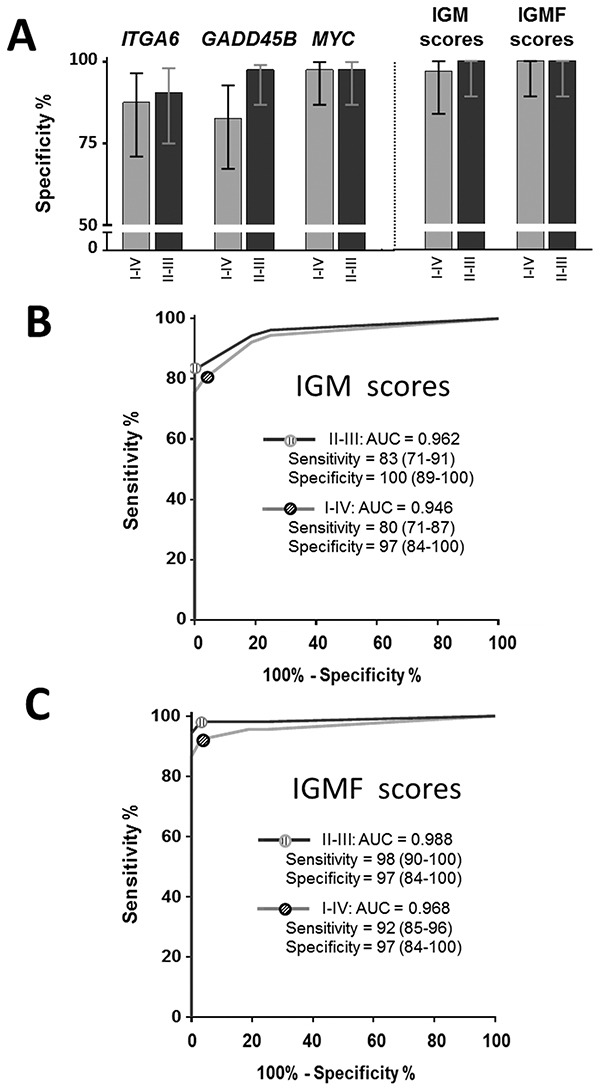
Use of the IGM (*ITGA6*, *GADD45B* and *MYC*) score for the diagnosis of CRC The IGM genes were evaluated individually and in combination by comparing specificities for 80% sensitivity in stage II-III and stage I-IV CRCs as well as the IGMF score, which combines IGM genes with iFOBT **A.** ROC curve analysis of the IGM and IGMF scores were determined for stage I-IV and II-III CRC **B, C.** Sensitivity and specificity provided in % (95% CI).

For the initial screening, we randomly selected 10 controls, 10 Ad and 20 stage II CRCs in order to identify genes that can lead to the detection of less advanced lesions. None of the tested markers showed a marked sensitivity for Ad but three of them, *GADD45B*, *MYBL2* and *MYC* showed a significantly increased expression in stool samples of patients with stage II CRC (Table 2). Further analyses of all samples confirmed that *GADD45B* and *MYC* were significantly increased in stools of patients with stage I-IV CRCs (P < 0.0001). IGM score was thus calculated as the sum of the ranking scores (calculated as above) of the three markers (*ITGA6*, *GADD45B* and *MYC*). For a fixed 80% sensitivity, specificities, which in CRCs were all above 83% individually, increased to 97% with the IGM score (Figure 3A). ROC analysis of the IGM score revealed an AUC of 0.95 (P < 0.0001) for the prediction of stage I-IV CRC vs controls and of 0.96 (P < 0.0001) for stage II-III CRC vs controls (Figure 3B). Interestingly, as expected from the data obtained with the IF scores, combining the iFOBT data with the IGM score to generate the IGMF score led to a significant improvement of the detection of both stage I-IV and stage II-III CRCs (Figure 3C).

**Table 2:** Screening for additional mRNAs markers

Genes^1^	Controls^2^	CRC II	P Value*
GADD45B	375 (233-787)	13,183 (3,595-56,334)	< 0.0001
MYBL2	0.2 (0.2-0.2)	39.2 (0.2-99.0)	< 0.01
MYC	11.8 (11.8-11.8)	2,265 (985-15,861)	< 0.0001
MKI67	BDL	BDL	

1 Initially selected on the basis of their expression in primary tumors in association with CRC recurrence (Oncotype)

2 Data presented in copy number as median (interquartille range) in thousands

* Kruskal-Wallis test was used to compare CRC II to control samples (n=10).

BDL: Below detection level

## DISCUSSION

The data from this study demonstrate that mRNAs can be used advantageously as valid targets for the setting of a sensitive and specific colon cancer stool screening assay. In this work, we used *ITGA6*, a well-characterized integrin transcript up-regulated in a majority of colorectal tumors [26, 27] as proof of concept using distinct approaches.

First, by evaluating the copy number of *ITGA6* in stool samples, we showed that the transcript can be detected at significant levels in all colorectal lesions with an overall sensitivity and specificity of 81 and 88% for CRCs and of 75 and 88% for Ad. *ITGA6* detection in stools is consistent with its over-expression in tumor cells [26, 27]. The significant expression of this transcript is thus a strong indicator of the presence of intact cells in stools of patients with intestinal lesions. While factors contributing to the detection of exfoliated colonic epithelial cells in neoplasia have been analyzed in detail [20], the potential to detect exfoliated cells from patients with cancer vs controls may rely on the cells' potential to resist anoikis [31], in contrast to normal cells in which disruption of cell-matrix interactions induces apoptosis [32], as suggested by Ahlquist et al. [21] and others [22, 23]. In this context, the particular sensitivity of mRNA to degradation in a hostile environment makes this molecule an attractive target for CRC screening. The 5-10 times increase in mRNA levels of *ITGA6* in stools of patients with intestinal lesions may thus be attributed to the higher survival of exfoliated cancer and pre-cancer cells in the stools (and their mRNAs) as compared with normal cells.

Second, we assessed *ITGA6A*, a variant of *ITGA6* encoding the pro-proliferative integrin α6A subunit that is up-regulated in a majority of CRC and is responsible for the overall increase of *ITGA6* in primary tumors [26, 27]. Interestingly, *ITGA6A* expression is restricted to the crypt in the control intestine thus not present in normal exfoliated cells but present in the majority of CRC cells [26], thus likely to be released in the stools. As expected, *ITGA6A* was detected at low levels in controls and Ad while its relative amount in samples from stage II and III CRCs represented approximately half of the total *ITGA6* copies in these samples, an observation consistent with our previous observations of the up-regulation of the *ITGA6A*/*ITGA6* ratio in these primary CRC lesions [27]. The results indicate that genes specifically expressed in tumor cells may present an additional value in the setup of a stool mRNA screening assay.

Third, we evaluated the possibility that combining cell component-based assays with a fecal hemoglobin test could improve overall sensitivity, as shown for the stool DNA test [33], since both groups detect independent events. The latter is well illustrated by the high level of rescue observed in the identification of iFOBT negative lesions with *ITGA6*. As expected from this complementarity, combining the data from the iFOBT test with *ITGA6* in an algorithm referred to as the IF score showed significant improvement in the overall sensitivity for both Ad and CRC as compared with ITGA6 alone.

Finally, one of the strengths of qPCR-based analysis relies on the fact that multiplex amplification of multiple target genes is more and more becoming a standard procedure in clinical diagnostic laboratories and does not require significant additional labor as compared with non-targeted qPCR, the more time consuming steps being mRNA extraction and preparation for qPCR. As a proof of concept for the set-up of an enhanced mRNAs assay, we screened for markers previously shown to be linked to stage II and III CRC recurrence risk [34]. Two of them were detected in stool samples of CRC patients with sensitivities and specificities > 80%: *GADD45B* and *MYC*. Combined with *ITGA6* as the IGM score, they allowed an increase in the specificity of up to 97% while in combination with the iFOBT, sensitivity and specificity for the detection of lesions were at 92 and 97% for stage I-IV CRC and 98 and 97% for stage II-III CRC.

Taken together, the results of this study compare advantageously with those from other gene-based screen stool assay studies for the detection of colorectal cancer. Indeed, a number of studies have reported the detection of various mRNA such as MMP7, PTGS2, MYBL2 and TP53 at higher levels in samples of patients with CRC as compared with those of healthy volunteers [18, 20, 23]. However, sensitivities were found to be quite variable depending on the mRNA source and isolation method as well as optimization of PCR conditions. For instance, reported sensitivities for PTGS2 (COX-2) and MMP7 varied from 34 to 90% and 31 to 65%, respectively, depending on the study [17, 18, 20, 23, 35].

In conclusion, these data based on the stool detection of *ITGA6* confirm the usefulness and reliability of mRNAs assays. Considering the automation of mRNA preparation in the clinic and the potential of qPCR, the validation of additional candidate genes specific for colorectal lesion detection including adenomas and CRC at all stages and their analysis by multiplex qPCR represents a powerful and robust approach that can be combined with iFOBT for improving the detection of colorectal lesions at a curable stage, an important criteria for CRC screening [3]. While the impact of this proof-of-concept study is somewhat limited by the number of genes included and the fact that analyses were performed on archived specimens, we think that these encouraging findings should lead to further studies on large asymptomatic populations.

## MATERIALS AND METHODS

### Patients and samples

Samples in this study were collected from patients and controls from the Hamamatsu University School of Medicine. All patients and subjects provided written informed consent. The study was approved by the institutional research ethics committees of the Hamamatsu University School of Medicine and the Centre Hospitalier Universitaire de Sherbrooke. The study cohort included 91 patients with stage I-IV CRC (24 stage I; 32 stage II; 22 stage III, and 13 stage IV), as well as 24 with advanced adenomas (Ad; defined as ≥ 1 cm at the greatest dimension) which were diagnosed both colonoscopically and histologically. A total of 60 patients displaying no pathological findings were used as control subjects. Characteristics of patients and lesions are provided in Table 3. Stool samples were either collected before colonoscopy from all control patients and patients with adenomas as well as a subset of patients with CRC (∼ 10%) or 2-4 weeks after colonoscopy and biopsy, for patients with CRC diagnosed colonoscopically in the outpatient unit, but before surgery or endoscopic resection [17]. Samples were stored at 4°C immediately after collection and transferred within 6 h to −80°C for storage of up to 2 years before RNA extraction.

**Table 3:** Characteristics of patients and lesions

	CRC	Ad	Controls
n	91	24	60
Age (y), median (range)	69 (27-86)	66 (33-87)	59 (26-76)
Sex			
Female : male	32 : 59	11 : 13	16 : 44
Lesion site			
Proximal^1^	36	10	
Distal^2^	55	14	
Stage^3^			
I	24		
II	32		
III	22		
IV	13		

1 Caecal, ascending and transverse colon lesions

2 Descending, sigmoid and rectal lesions

3 Staging according to the TNM classification of tumors

### Immunochemical FOBT

Quantification of hemoglobin in stools using a single immunochemical fecal occult blood test (iFOBT) with MagStream HemSp (Fujirebio Inc., Tokyo, Japan) was performed on all patients and controls. The iFOBT tests were performed in a clinical laboratory of the hospital independent of the authors based on a cutoff level set at 20 ng/ml (80 μg/g) as suggested by the manufacturer. Using a subset of samples similar to those tested in the present study, the overall iFOBT sensitivity and specificity reported were 73% (95% CI: 60-83%) and 90% (95% CI: 73-98 %), respectively [18].

### RNA isolation, reverse transcription and PCR amplification

RNA was isolated from fecal specimens as described previously [17, 20]. cDNA was synthesized using M-MLV Reverse Transcriptase, RNase H Minus (Takara Bio Inc., Otsu, Japan) with 0.375 μg total RNA from stools and 750 ng random hexamers in a final reaction volume of 60 μl.

For quantitative real-time PCR (qPCR), commercially available TaqMan primers and probe mixtures were used as described before [20]. TaqMan Gene Expression Assays (Applied BioSystems, Foster City, CA) consisting of a pair of unlabeled primers and a TaqMan probe labeled with FAM at the 5′ end were used for amplification of *ITGA6* (Hs01041011_m1) and *B2M* (Hs00984230_m1), following the manufacturer's instructions. Briefly, the reaction mixture included 10 μl of TaqMan Master Mix (Applied Biosystems), 1 μl of a 20x TaqMan primer and probe mixture and 2 μl of cDNA in a total reaction mixture of 20 μl. qPCR was performed for 60 cycles of 30 sec 95°C, 1 min 60°C in a Mx3000P Real Time PCR machine (Stratagene, Mississauga, Ontario).

### Preamplification and qPCR

For preamplification, the TaqMan PreAmp Master Mix Kit (Life Technologies) was used to provide unbiased, multiplex preamplification of specific amplicons for analysis with TaqMan gene expression assays. Commercially available TaqMan primer and probe mixtures were used for the amplification of the following selected genes: *ITGA6* (Hs01041011_m1), *B2M* (Hs00984230_m1), *ITGA6A* (Hs01041013_m1), *GADD45B* (Hs00169587_m1), *MYBL2* (Hs00942543_m1), *MYC* (Hs00153408_m1) and *MKI67* (Hs01032434_m1). Briefly, 20x TaqMan gene expression assays were pooled at a final concentration of 0.2x in 1x TE, combined with 5 μl fecal cDNA and 2x TaqMan PreAmp Master Mix in a total volume of 20 μl, then preamplified for 14 PCR cycles of 15 seconds at 95°C, 4 minutes at 60°C. Preamplification products were diluted 1:20 with 1x TE Buffer, aliquoted and stored at −80°C.

qPCR reactions were prepared by combining 5 μl of 20x diluted preamplification products with 10 μl TaqMan Gene Expression Master Mix and 1 μl of each 20x TaqMan Gene Expression Assay in a total volume of 20 μl. qPCR was performed in the Mx3000p system as above.

### Data presentation and statistical analysis

Stool mRNA detection results were presented as copy number. For each gene, a standard reference curve was generated using serial 5 fold dilutions of a cDNA stock solution of the target sequence quantified using a NanoDrop 1000 spectrophotometer (NanoDrop, Wilmington, DE).

For statistics, data were analyzed using Prism 6 software (GraphPad). Correlations of stool mRNA detection by qPCR from preamplified vs non-preamplified samples were evaluated using the nonparametric Spearman correlation test. Comparisons of mRNA expression in stool samples from controls and patients with lesions were expressed as median with interquartile range and analyzed by the Kruskal-Wallis test followed by Dunn's multiple comparison test. Areas under the receiver operating characteristic (ROC) curves were calculated using Prism 6. Sensitivities and specificities were expressed in % with a 95% confidence interval (CI). Optimal cutoff values were calculated with Cutoff Finder [36]. Statistical significance was defined as *P* < 0.05.
